# Co-silencing of *PhENO1* and *PhPPT* alters anthocyanin production by reducing phosphoenolpyruvate supply in petunia flower

**DOI:** 10.1093/hr/uhaf040

**Published:** 2025-02-11

**Authors:** Xin Li, Jiahao Cao, Guiyun Jiang, Wenqi Deng, Huimin Deng, Weiyuan Yang, Yixun Yu, Juanxu Liu

**Affiliations:** College of Horticulture, South China Agricultural University, Guangzhou 510642, China; College of Horticulture, South China Agricultural University, Guangzhou 510642, China; College of Horticulture, South China Agricultural University, Guangzhou 510642, China; College of Horticulture, South China Agricultural University, Guangzhou 510642, China; College of Horticulture, South China Agricultural University, Guangzhou 510642, China; College of Horticulture, South China Agricultural University, Guangzhou 510642, China

## Abstract

The shikimate pathway is crucial for the production of aromatic amino acids and various secondary plant products, including anthocyanins. Phosphoenolpyruvate (PEP) is an important source for shikimate production. The pre-chorismate part of the shikimate pathway is confined to plastids. There are three sources of PEP in plastids. PEP can be imported into the plastids from cytoplasm via the PEP/phosphate translocator (PPT), and it can also be generated in plastids via enolase (ENO) and pyruvate orthophosphate dikinase (PPDK) catalysis. A large number of anthocyanins are synthesized in the flowers of most ornamental plants in the coloring stage. However, the source of PEP, the precursor of anthocyanin synthesis, is still unknown. Herein, *Petunia hybrida PhENO1*, *PhPPT* and *PhPPDK* genes were identified and their expression patterns and subcellular localization of encoded proteins were analyzed. Silencing of *PhENO1*, *PhPPT*, and *PhPPDK* alone and co-silencing of *PhENO1* and *PhPPDK* or *PhPPT* and *PhPPDK* did not exhibit any visible phenotypic change compared with the control, while co-silencing of *PhENO1* and *PhPPT* resulted in the flower color change from purple to light purple. The content of PEP, shikimate, flavonoids, anthocyanins, and aromatic amino acids were all significantly decreased in *PhENO1* and *PhPPT* co-silenced plants. Co-silencing of *PhENO1* and *PhPPT* did not affect the expression level of key genes in anthocyanin synthesis and shikimate pathways. Furthermore, co-silencing of *PhENO1*, *PhPPT*, and *PhPPDK* resulted in a phenotype similar to the co-silencing of *PhENO1* and *PhPPT*. Altogether, our study suggested that PEP used for anthocyanin synthesis is mainly provided by PhENO1 and PhPPT, rather than PhPPDK.

## Introduction

The shikimate pathway in plants serves as a critical junction between primary and secondary metabolism, with its initiation confined to the stroma of plastids [5, 6]. Glucose is metabolized into phosphoenolpyruvate (PEP) via glycolysis and converted into erythrose-4-phosphate (E4P) through the pentose phosphate pathway. PEP and E4P are two precursors of the shikimate pathway, and they both enter the shikimate pathway through the catalysis of 3-deoxy-D-arabino-heptulosonate 7-phosphate synthase (DAHPS) [5, 7, 8] (Supplementary Fig. S1).

PEP functions as a central intermediate in both prokaryotic and eukaryotic metabolism and can be obtained via three ways (Supplementary Fig. S1; [1, 2]) PEP is generated in plastids via the complete glycolytic pathway catalyzed by phospho-glyceromutase (PGyM) and enolase (ENO). The conversion of 3-phosphoglycerate (3PGA) to 2-phosphoglycerate (2PGA) is facilitated by PGyM, followed by the conversion of 2PGA to PEP, catalyzed by ENO1 [9, 10]. [3] In the cytoplasm, PEP is transported into plastids via the phosphoenolpyruvate/phosphate translocator (PPT) [11, 12]. [4] Pyruvate can be converted to PEP in plastids by pyruvate orthophosphate dikinase (PPDK) [13, 14, 15], and this reaction is reversible; PEP can also be converted back to pyruvate through the catalysis of plastidial pyruvate kinase (PK) (Ambasht and Kayastha, 2002; [16]).

ENO1 facilitates the dehydration of 2PGA to PEP within the glycolytic pathway. Nowadays the enolases have been cloned from many species, including *Arabidopsis thaliana* [9]*, Nicotiana tabacum* [10]*, Zea mays* [17], and *Mesembryanthemum cristallinum* [18]. *A. thaliana ENO* family contained three members, AtENO1 (At1g74030) is localized to the plastid, while AtENO2 (At2g36530) and AtENO3 (At2g36530) are localized to the nucleus and cytoplasm [9]. *AtENO1* is highly expressed in roots and young siliques, but *AtENO1* mRNA is virtually absent from mature leaves and flowers [9]. The process of transforming 3PGA into PEP through the glycolytic pathway is lacking in chloroplasts and most nongreen plastids, and this is mainly due to the low or no expression of *AtPGyM* and At*ENO1* in these locations [2, 4, 10, 19]. The *eno1* mutant of *A. thaliana* exhibited distorted trichomes and fewer root hairs [9]. Enolase antisense plants in *N. tabacum* display growth retardation and a reticulate leaf phenotype. A decrease in enolase activity affects secondary pathways like the shikimate pathway and a branch of amino acid biosynthesis [10].

PEP can also be imported from the cytosol into the plastid through a specific translocator, PPT. There are two *PPT* members in *A. thaliana*, *AtPPT1* (At5g33320) and *AtPPT2* (At3g01550). AtPPT1 and AtPPT2 are localized in chloroplasts and exhibit similar substrate specificity, while *AtPPT1* and *AtPPT2* display different expression patterns [20]. The role of *PPT* in transporting PEP to plastids for the synthesis of aromatic amino acids (AAAs) has been well established in *A. thaliana* [12, 21], *Vitis vinifera* [22], *Brassica napus* [23], *Mesembryanthemum crystallinum* [24], *Galdieria sulphuraria* [25], and *Nitzschia* sp. [26]. Research has demonstrated that the *cue1* mutant of *A. thaliana*, characterized by reduced expression of *AtPPT1*, displays a reticulate leaf phenotype, featuring dark green paraveinal areas and light green interveinal regions [21, 27]. Furthermore, the *cue1* mutant shows deficiencies in both chloroplast and mesophyll development [21, 27]. *A. thaliana ppt2* mutant only showed a slightly retarded growth compared with the wild type [28]. Deletion of *VviPPT1* resulted in a noticeable phenotype characterized by reduced anthocyanins and a decrease in several primary and secondary metabolites, including shikimate, phenolic compounds, and their derivatives [22].

PPDK catalyzes the conversion of pyruvate to PEP. The biological role of PPDK was described in *Oryza sativa* [29], *Z. mays* [15], and *A. thaliana* [30]. In *A. thaliana*, AtPPDK is encoded by one gene with two distinct promoters, forming two different transcripts [14]. The ectopic expression of *PPDK* restored the phenotype of the *cue1* mutant [12], suggesting the involvement of PPDK in providing PEP to the shikimate pathway [31].

The shikimate pathway finally forms chorismate, the final common precursor of AAAs, including phenylalanine (Phe). The phenylalanine synthesis pathway occurs through two pathways, the arogenate pathway (occur in plastid) and phenylpyruvate pathway (occur in cytoplasm) [32]. In the arogenate pathway, chorismate undergo catalysis by chorismate mutase 1 (CM1), prephenate aminotransferase (PPA-AT), and arogenate dehydratase (ADT) to ultimately produce Phe. Subsequently, Phe is transported from the plastid to the cytoplasm through the plastidial cationic amino acid transporter (pCAT). In the phenylpyruvate pathway, chorismate are catalyzed by prephenate dehydratase (PDT) and phenylpyruvate aminotransferase (PPY-AT) to produce Phe in the cytoplasm (Supplementary Fig. S1). Phenylpropanoids, derived from Phe, constitute one of the most diverse classes of plant secondary metabolites, including flavonoids, anthocyanins, tannins, and lignin [1, 33]. Phe is an essential precursor of anthocyanins. Large amounts of anthocyanins, which are provided through the shikimate pathway, are synthesized during flower coloring stage in numerous ornamental plants. However, the source of PEP required for anthocyanins synthesis is still unknown. Petunia (*Petunia hybrida*), an important ornamental plant, is a model plant for studying anthocyanin synthesis. In this study, we elucidate the roles of *PhENO1*, *PhPPT*, and *PhPPDK* in providing the requisite PEP for anthocyanins synthesis. We found that individual silencing of these genes, as well as the co-silencing of *PhENO1* and *PhPPDK* or *PhPPT* and *PhPPDK*, did not lead to discernible phenotypic changes compared to the control. Conversely, the co-silencing of *PhENO1* and *PhPPT* resulted in alterations in flower color and a significant decrease in total anthocyanin content compared with the control. Moreover, the co-silencing of *PhENO1*, *PhPPT*, and *PhPPDK* produced a phenotype similar to that of co-silencing of *PhENO1* and *PhPPT*. The levels of PEP, shikimate, Phe, and flavonoids in pTRV2-PhENO1-PPT-treated plants were significantly decreased compared with the control. These findings indicate that *PhENO1* and *PhPPT* jointly provide the necessary PEP source for anthocyanin synthesis in petunia.

## Results

### Identification of *PhENO*, *PhPPT,* and *PhPPDK* gene families in petunia

**Figure 1:**
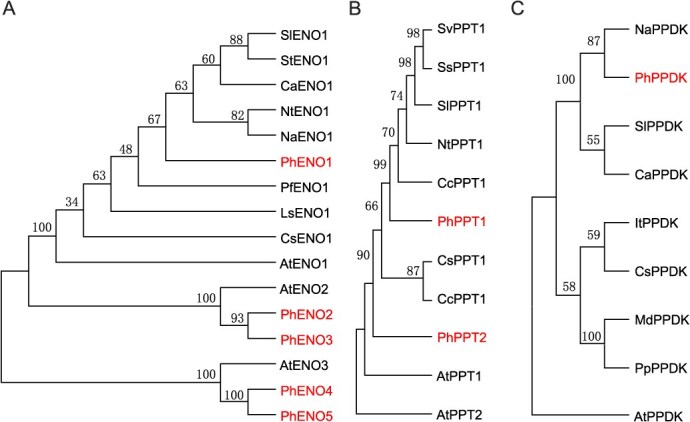
Molecular phylogenic tree of ENO (A), PPT (B), and PPDK (C) family. A ML phylogenetic tree was constructed by MEGAX with 1000 bootstrap replicates. Detailed information of all proteins was showed in Supplementary Table S5.

To explore the function of *PhENO* in petunia, a basic local alignment search tool (BLAST) search was conducted on the petunia genome, leading to the identification of five PhENO genes named PhENO1-5. The predicted proteins of PhENO1-5 comprised 480, 469, 429, 409, and 223 amino acids, with calculated molecular masses of 51.22, 50.71, 46.41, 44.80, and 24.46 kDa, correspondingly (Supplementary Fig. S2). A maximum likelihood (ML) phylogenetic trees showed that the PhENO family proteins are categorized into three groups. PhENO1 and AtENO1 were clustered in the same group, indicating that PhENO1 is the homologue of AtENO1 in petunia (Fig. 1A). All members of the PhENO family contain a conserved domain of PLN00191 subfamily (Supplementary Fig. S5C).

Similarly, through the BLAST search, we identified two members of PhPPT family in petunia. Both PhPPT1 and PhPPT2 contain a sugar-phosphate transporter domain (Supplementary Fig. S3). Phylogenetic analysis demonstrated a close evolutionary relationship between two PhPPTs and AtPPT1 (Fig. 1B). In addition, we discovered that *PhPPDK* is a single-copy gene family in petunia. The predicted protein of PhPPDK consists of 968 amino acids and has a calculated molecular mass of 106.08 kDa (Supplementary Fig. S4). Similar to other PPDKs, PhPPDK contains a PEP-utilizing enzyme site in N-terminus and three conserved domains (Supplementary Fig. S5H). PhPPDK is most closely related to NaPPDK, while AtPPDK is most distantly related to PhPPDK (Fig. 1C).

### Subcellular localization of PhENO1, PhPPT1, PhPPT2, and PhPPDK

To gain further insight into the functions of PhENOs, PhPPTs, and PhPPDK, we first predicted the cellular localization using the online software Cell-PLoc 2.0 [34]. The results indicated that PhENO1, PhPPT1, PhPPT2, and PhPPDK are localized in plastids, whereas the other four members of the PhENO family are localized in cytoplasm.

**Figure 2:**
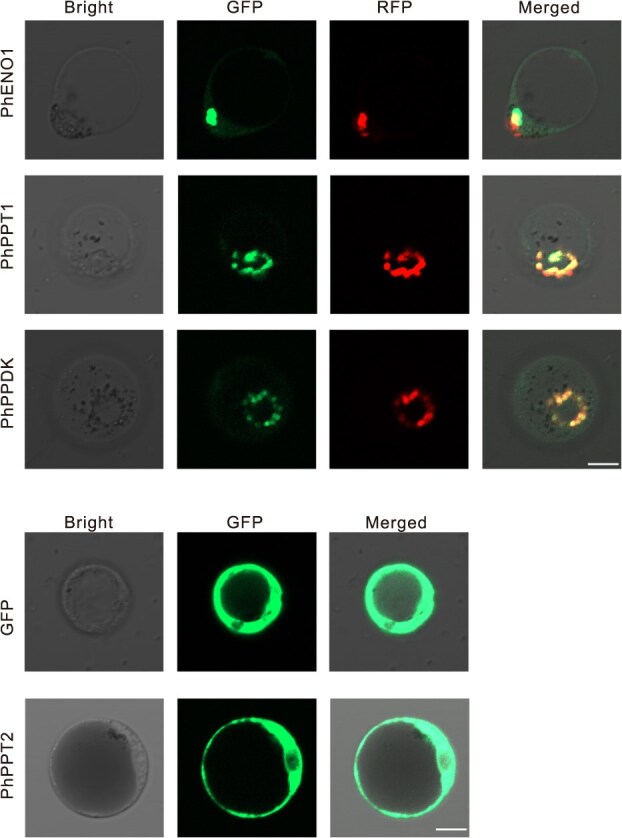
Subcellular localization of free-GFP, PhENO1, PhPPT1, PhPPT2, and PhPPDK in petunia corolla protoplasts with the plastid localized RFP marker. Bars = 10 μm.

We further investigated the subcellular localization of PhENO1, PhPPT1, PhPPT2, and PhPPDK in petunia cells through experiments. The vectors 35S: GFP, 35S:PhENO1: GFP, 35S:PhPPT1: GFP, 35S:PhPPT2: GFP, and 35S: PhPPDK: GFP were constructed and subsequently transformed into petunia corolla and leaf protoplasts with the plastid localized RFP marker [35]. The fusion proteins were visualized using a laser-scanning confocal microscope. The results indicated that PhENO1, PhPPT1, and PhPPDK are localized in the plastids, whereas PhPPT2 is localized in the cytoplasm in both corolla and leaf protoplasts (Fig. 2; Supplementary Fig. S7).

### Expression patterns of *PhENO1*, *PhPPT1, PhPPT2,* and *PhPPDK*

**Figure 3:**
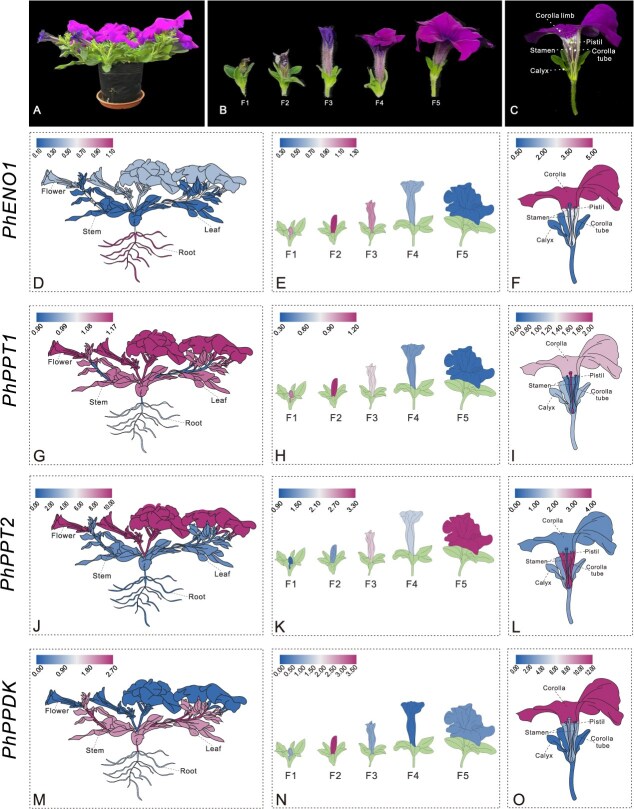
A series of fancy heatmaps displaying the expression patterns of *PhENO1*, *PhPPT1, PhPPT2,* and *PhPPDK* determined using qPCR. (A) Photograph of petunia growing in pots. (B) Illustrations of the five stages of flower development (C) Diagram illustrating the structure of a petunia flower. Expression of *PhENO1* (D), *PhPPT1* (G), PhPPT2 (J), and *PhPPDK* (M) in various organs (root, stem, leaf, and flower). Expression of *PhENO1* (E), *PhPPT1* (H), *PhPPT2* (K), and *PhPPDK* (N) at different stages of flower development. Flower development stages are defined as F1 (0.5 cm length), F2 (1.0 cm), F3 (2.0 cm, the coloring stage), F4 (3.0 cm), and F5 (flowering stage). Expression of *PhENO1* (F), *PhPPT1* (I), *PhPPT2* (L), and *PhPPDK* (O) in different flower parts (corolla limb, corolla tube, stamen, pistil, and calyx) at stage F5. *Cyclophilin* (accession no. EST883944) was used as the internal reference gene for quantifying cDNA abundance. The data are presented as the mean ± SD (*n* = 3). Statistical analysis was conducted using one-way analysis of variance (ANOVA) followed by DMRT with three biological replicates.

Using *PhCYP* (accession no. EST883944) as an internal reference gene, the expression levels of *PhENO1*, *PhPPT1*, *PhPPT2*, and *PhPPDK* were examined in different plant organs (root, stem, leaf, and flower), five development stages of flowers, and different tissues of flower at stage F5 in petunia ‘Ultra’. Among different organs in petunia, *PhENO1*, *PhPPT1*, *PhPPT2,* and *PhPPDK* exhibited the highest expression level in roots, flowers, flowers, and stems, respectively (Fig. 3D, G, J, and M; Supplementary Fig. S8A, D, G, and J). Across the five developmental stages of flowers, the expression levels of *PhENO1*, *PhPPT1,* and *PhPPDK* were highest at the F2 stage (Fig. 3E, H, and N and Supplementary Fig. S8B, E, and K), displaying an initial increase followed by a decline. Conversely, the expression of *PhPPT2* steadily rose from the F1 to F5 stage, reaching its highest point at F5 (Fig. 3K and Supplementary Fig. S8H). Petunia corolla limb has the darkest color at the F3 stage, while the peak expression levels of these three genes were at F2 stage, slightly preceding F3 (Fig. 3B), suggesting a potential role for *PhENO1*, *PhPPT1*, and *PhPPDK* in flower coloration. Among the five tissues of flower (calyx, corolla limb, corolla tube, pistil, and stamen), *PhENO1*, *PhPPT1*, *PhPPT2,* and *PhPPDK* exhibited the highest expression level in the corolla, stamens, corolla tube, and corolla limb, respectively (Fig. 3F, I, L, and O and Supplementary Fig. S8C, F, I, and L).

### VIGS-mediated co-silencing of *PhENO1* and *PhPPT* alters flower color and reduces total anthocyanin content

Based on the analysis of protein homology, subcellular localization and genes expression, *PhENO1*, *PhPPT1*, *PhPPT2*, and *PhPPDK* are potentially involved in supplying the necessary PEP for anthocyanin synthesis.

To investigate the roles of *PhENO1*, *PhPPT1*, *PhPPT2,* and *PhPPDK* in flower color formation, pTRV2 derivative vectors were constructed to suppress the expression of these genes using the highly efficient VIGS system we have already established in petunia ‘Ultra’ [36, 37]. Given the high sequences similarity between *PhPPT1* and *PhPPT2*, 300 bp fragments from their conserved regions were selected to construct the pTRV2-PhPPT vector for silencing both genes. Similarly, approximately 300 bp fragments from the 3′-untranslated region of *PhENO1* and *PhPPDK* were inserted into the pTRV2 vector to generate pTRV2-PhENO1 and pTRV2-PhPPDK vectors, respectively. The pTRV2-GFP vector, previously engineered, was used as a control. Each group consisted of thirty to thirty-five petunia plants for the infection.

**Figure 4:**
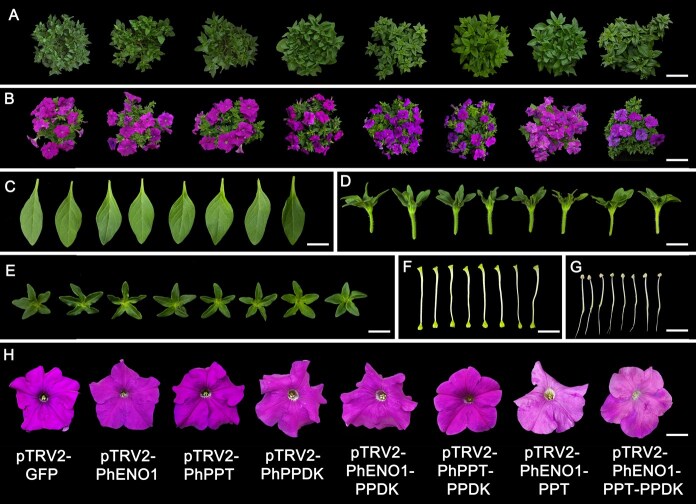
Phenotypic characteristics of *PhENO1*, *PhPPT,* and *PhPPDK (co-)silenced plants*. The images depict the following (A-H) from left to right: pTRV2-GFP (control) and pTRV2-PhENO1, pTRV2-PhPPT, pTRV2-PhPPDK, pTRV2-PhENO1-PPDK, pTRV2-PhPPT-PPDK, pTRV2-PhENO1-PPT, and pTRV2-PhENO1-PPT-PPDK-treated plants: top view of vegetative growth stage (A) and blooming stage (B), leaves (C), side (D) and top (E) view of calyx, pistils (F), stamens (G) and flowers (H). The scale bars are as follows: 15 cm in (A) and (B), 2.5 cm in (C), 1.5 cm in (D), 2 cm in (E), 1 cm in (F) and (G), and 3 cm in (H).

**Figure 5:**
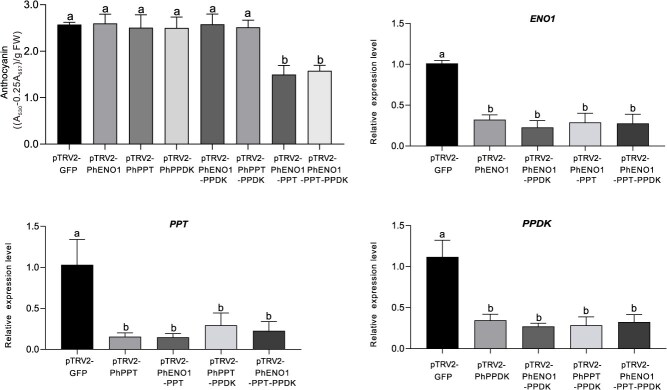
The impact of pTRV2-PhENO1-PPT treatment on the total anthocyanin content in petunia flower (A) and the expression of *PhENO1* (B), *PhPPT* (C), and *PhPPDK* (D) in plants treated with the respective vectors. The data are displayed as mean ± SD (*n* = 9).

Approximately, one month after infection, we observed no significant phenotypic changes in *PhENO1*, *PhPPT,* and *PhPPDK* alone silenced plants compared to the control. Given that silencing the possible PEP-providing genes individually did not markedly alter the petunia phenotype, we hypothesized that two or all three pathways may be involved in providing PEP for flower color formation. We subsequently developed four co-silencing vectors: pTRV2-PhENO1-PPT, pTRV2-PhENO1-PPDK, pTRV2-PhPPT-PPDK, and pTRV2-PhENO1-PPT-PPDK. The results indicated that pTRV2-PhENO1-PPDK and pTRV2-PhPPT-PPDK-treated plants exhibited no changes compared with the control (Fig. 4A-G). In contrast, pTRV2-PhENO1-PPT-treated plants displayed a lighter flower color compared to the control (Fig. 4H), the total anthocyanin content significantly decreased by 41.63% (Fig. 5A). Aside from the change in flower color, there were no other visible differences in pTRV2-PhENO1-PPT-treated plants compared to the control (Fig. 4A-G). Furthermore, pTRV2-PhENO1-PPT-PPDK-treated plants exhibited a phenotype similar to that of the pTRV2-PhENO1-PPT-treated plants. We assessed the expression level of *PhENO1*, *PhPPT*, and *PhPPDK* in vectors-treated plants using qPCR, confirming that the expression of these genes was inhibited in the respective treated plants (Fig. 5B, C, and D. The degree of decrease in *PhENO1* and *PhPPT* expression levels in leaves were significantly lower than in flowers (Supplementary Fig. S9). Collectively, these findings suggest that PEP utilized for anthocyanin synthesis is primarily derived from PhENO1 catalysis and PhPPT transport rather than from PhPPDK catalysis. Therefore, we will focus our future studies on the pTRV2-PhENO1-PPT-treated plants and the control plant.

### Co-silencing of *PhENO1* and *PhPPT* results in a reduction of PEP, shikimate, flavonoids, and aromatic amino acid content in petunia

**Figure 6:**
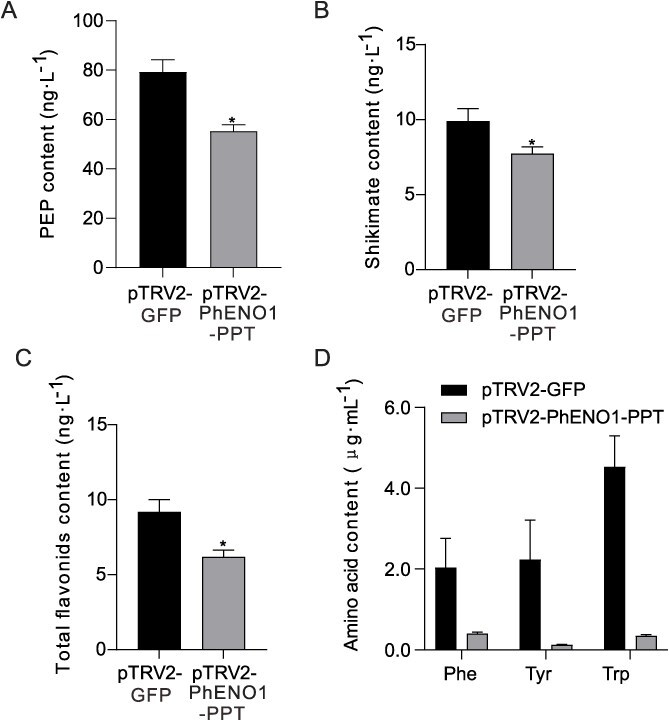
The levels of PEP (A), shikimate (B), flavonoids (C), and amino acids (D) in pTRV2-PhENO1-PPT-treated plants. Data are presented as mean ± SD (*n* = 3). An asterisk indicates a statistically significant difference at *P* < 0.05.

We examined the levels of PEP, shikimate, and flavonoids in pTRV2-PhENO1-PPT-treated plants. These results demonstrated a notable decrease in PEP, shikimate, and flavonoid concentrations by 30.16%, 24.82%, and 32.55%, respectively, compared to the control group (Fig. 6A–C). These results suggest that *PhENO1* and *PhPPT* are involved in providing the source of PEP for shikimate production.

AAAs are synthesized through the shikimate pathway [12]. We further investigated the impact of co-silencing of *PhENO1* and *PhPPT* on AAAs level. The results revealed a substantial reduction in the contents of phenylalanine (Phe), tryptophan (Trp), and tyrosine (Tyr) by 80.14%, 96.70%, and 94.30%, respectively, in pTRV2-PhENO1-PPT-treated plants compared to the control group (Fig. 6D).

### Investigation of expression levels of pivotal genes in the shikimate pathway, phenylalanine pathway, and anthocyanin pathway in pTRV2-PhENO1-PPT-treated plants

We evaluated the expression levels of six pivotal genes (*PhDAHPS*, *PhDHQS*, *PhDHQSDH*, *PhSK*, *PhEPSPS1*, and *PhCS*) in the shikimate pathway in pTRV2-PhENO1-PPT-treated plants using a qPCR assay (Supplementary Figs S1 and S10). These results showed that the co-silencing of *PhENO1* and *PhPPT* did not impact the expression levels of these pivotal genes in the shikimate pathway.

Subsequently, we analyzed the expression levels of pivotal genes in the phenylalanine biosynthesis pathway in pTRV2-PhENO1-PPT-treated plants. We found that the genes in the arogenate pathway (*PhCM1*, *PhPPA*-AT, *PhADT1*, and *PhpCAT*) showed a downward trend in pTRV2-PhENO1-PPT-treated plants, while most genes in the phenylpyruvate pathway (*PhCM2*, *PhADT2*, and *PhPPY-AT*) exhibited an upward trend (Supplementary Fig. S11).

Furthermore, we investigated the expression levels of eight pivotal genes (*PhPAL1*, *PhPAL2*, *PhPAL3*, *PhCHS*, *PhF3H*, *PhF3’5’H*, *PhANS,* and *Ph5GT*) in the anthocyanin biosynthesis pathway. The results showed that the expression levels of the pivotal genes in the anthocyanin biosynthesis pathway did not exhibit significant changes in pTRV2-PhENO1-PPT-treated plants compared to the control (Supplementary Fig. S12).

## Discussion

In plastids, the shikimate pathway initiates with a union of PEP and E4P, offering a pathway for the production of aromatic amino acids (AAAs) and secondary metabolites like anthocyanins [5]. Previous studies have highlighted the crucial roles of *ENO*, *PPT,* and *PPDK* in providing PEP in plastid (Supplementary Fig. S1). In this study, to explore the sources of PEP in plastids for anthocyanin synthesis, *PhENO1*, *PhPPT,* and *PhPPDK* were (co-)silenced. The co-silencing of *PhENO1* and *PhPPT* led to a shift in flower color from purple to light purple, accompanied by a significant reduction in the levels of PEP, shikimate, phenylalanine, flavonoids, and anthocyanins.

In *A. thaliana*, the ENO family comprises three members, with only AtENO1 localized to plastids. AtENO1 is highly expressed in young roots but is not detected in old leaves and flowers [9]. In our study, PhENO1 was identified as the most homologous protein to AtENO1. Notably, *PhENO1* is expressed in flowers, with particularly high expression in the corolla limbs, whereas AtENO1 is absent in flowering tissues. These findings suggested that *PhENO1* may play a more significant role in flower development compared to *AtENO1*. Two T-DNA insertion mutants of *A. thaliana*, designated *eno1*, displayed abnormal trichome morphology and a decreased number of root hairs, but displayed no evident macroscopic phenotype [9]. Similarly, in our study, silencing of *PhENO1* alone did not result in any noticeable phenotypic changes at the macroscopic level in petunia.

In petunia, similarly to *A. thaliana*, the PPT family consists of two members, *PhPPT1* and *PhPPT2*. Phylogenetic tree and gene structure analyses revealed that PhPPT1 and PhPPT2 exhibit higher homology with AtPPT1, rather than AtPPT2. In contrast to *A. thaliana*, both *PhPPT1* and *PhPPT2* display peak expression levels in flowers, indicating their potential crucial roles in flower development. The function of PPT has been documented in several plants, including horticultural crops, in recent studies. The *A. thaliana AtPPT1* functional deletion mutant *cue1* exhibited a reticulate leaf phenotype with dark green paraveinal and light green interveinal regions [28, 38, 39]. In *Vitis vinifera*, knocking out *VviPPT1* resulted in a significant decrease in anthocyanin content in callus [22]. Knocking out of *Brassica napus BnaPPT1* results in delayed growth, leaf yellowing, and a significant decrease in seed oil accumulation [40]. However, the silencing of *PhPPT* alone in petunia did not induce similar phenotypic alterations observed in *A. thaliana*, *V. vinifera*, or *B. napus*. Although these studies demonstrate the involvement of PPT in PEP production, the specific metabolic pathways in which PEP participates vary among different plant species. The *cue1* mutant phenotype is not simply caused by a general restriction of the shikimate pathway because of a defect in a PPT ([12]). The PEP transported by PPT1 to the plastid in *B. napus* is involved in fatty acid synthesis in seeds via the pyruvate pathway [40]. In *V. vinifera*, PEP supplied by *VviPPT1* provides serves as a precursor for the anthocyanin pathway in callus through the shikimate pathway [22]. These findings imply that while PPTs in different plant species may share the role of transporting PEP to plasmids, the reduction or absence of PEP transport by PPTs can result in diverse phenotypic changes, including no significant phenotypic changes, due to the involvement of PEP in many primary metabolic pathways.

In *A. thaliana*, the single gene *AtPPDK* generates two transcripts, with the longer variant encoding a chloroplast-targeted protein that exhibits high expression levels in both leaves and flowers [14]. In contrast, *PhPPDK* in petunia is a single-copy gene that may only have one transcript (Supplementary Fig. S6). Spatial–temporal expression analysis of *PhPPDK* revealed that it has the highest expression level in stems and relatively low-expression level in flowers, implying that *PhPPDK* may have a limited role in flower color formation. Furthermore, existing studies have demonstrated that PPDK provides PEP to plastid mainly exists in C4 plants, and there is no proof that C3 plants can produce PEP through PPDK [3]. Silencing of *PhPPDK* did not result in any phenotypic changes in petunia, which is consistent with its classification as a C3 plant [41]. This finding further reinforces the notion that a pathway mediating the transport of PEP to plastids via PPDK is absent in C3 plants.

Homozygous double mutants of *cue1* and *eno1* were unattainable in *A. thaliana* [39]. Additionally, homozygous *cue1* heterozygous *eno1* mutants (*cc*E*e*) exhibited impaired vegetative growth, abnormal flower development, and up to 80% seed abortion [39]. Meanwhile, the contents of PEP, three AAAs and flavonoid in *cc*E*e* mutant were significantly diminished compared with the wild type [39]. In our study, co-silencing of *PhENO1* and *PhPPT* led to light flower color, as well as a significant reduction in shikimate, Phe, and anthocyanins, which were consistent with those of the *cc*E*e* mutant. No anthocyanins were detected in *cc*E*e* mutants due to the extremely low anthocyanin content in *A. thaliana* flowers [39]. In grapes, the knockout of *VviPPT1* alone produced an obvious visual low anthocyanin phenotype in callus, with reductions in shikimate, PEP, Phe, and anthocyanins levels, highlighting the crucial role of *VviPPT1* in callus [22]. Moreover，the key enzymes of the shikimate and anthocyanin pathways were inhibited at the protein activity and/or gene expression level when the *VviPPT1* was knockout. In our study, silencing of *PhPPT* alone did not induce any visible phenotypic changes in petunia, indicating that PPT may not be the sole protein providing PEP for anthocyanin synthesis. Further studies we found that *PhENO1* and *PhPPT* supply collectively supply PEP for the anthocyanin synthesis pathway. These results underscore the differing roles of PPT in anthocyanin synthesis between petunia corollas and grape callus. In addition, unlike in grape, the majority genes in the shikimate pathway and the anthocyanin pathway did not exhibit significantly changes in expression levels in pTRV2-PhENO1-PPT plants, while key genes in phenylalanine pathway were altered. Within the phenylalanine pathway, genes encoding the plastid-localized proteins showed downregulation in expression in pTRV2-PhENO1-PPT plants, whereas genes encoding cytoplasm-localized proteins displayed upregulation, indicating distinct response patterns of these pathways to PEP reduction. Collectively, we hypothesize that the reduction of PEP is the primary factor leading to decreased anthocyanin levels, without affecting enzyme activity. In our study, we propose that the decrease in Phe content is attributable to the co-silencing of *PhENO1* and *PhPPT*, which results in a reduction in the precursor PEP, although the qPCR results showed that the genes involved in the cytosolic pathway of phenylalanine synthesis are upregulated in the corollas treated with pTRV2-phENO1-PPT. The alterations in the expression levels of key genes within the phenylalanine pathway may be a consequence of feedback regulation resulting from the decrease in Phe content induced by the reduction in PEP levels.

In this study, pTRV2-PhENO1-PPT-treated plants exhibited significantly lower levels of aromatic amino acids. These amino acids are also important in other developmental processes, but the flower color change is the only phenotypic change observed here. We speculate that the reduction of these amino acids mediated by the co-silencing of *PhENO1* and *PhPPT* in vegetative organs could not be sufficient to cause visible phenotypic changes. In flowers, the reduction of these amino acids could prioritize meeting the basic growth and development needs of flower organs, while the synthesis of anthocyanins, which are not the most important for plants, is first affected.

We then delved into the specific mechanism of *PhENO1* and *PhPPT* involvement in PEP formation. In tobacco, antisense *NtENO1* led to a high level of 2PGA [10]. In the case of individual inhibition of PhENO1 expression, a decrease in *PhENO1* expression leads to a reduction in PEP biosynthesis from plastid. Given that ENO1 and PPT share a common precursor (2PGA), an increase in the precursor for PPT leads to the transportation of 2PGA to the cytoplasm by PhPPT, where it is converted into PEP. This suggests that the overall PEP content may remain stable. Conversely, when the expression of *PhPPT* is also inhibited, insufficient PPT prevents the transfer of 2PGA to the cytoplasm for PEP synthesis. Therefore, PhENO1 might have more 2PGA available for PEP synthesis, allowing the increased PEP to compensate for the deficiency resulting from *PhPPT* silencing. Hence, a reduction in PEP content is only noticeable when both *PPT* and *ENO1* expressions are simultaneously inhibited.

In conclusion, the findings of this study propose that *PhENO1* and *PhPPT*, rather than *PhPPDK*, play a role in supplying the PEP required for anthocyanin synthesis in petunia.

## Materials and methods

### Plant materials

The petunia we chose for experiment was *Petunia hybrida* ‘Ultra’. *P. hybrida* ‘Ultra’ plants were grown under controlled conditions [37] from sowing to subsequent experiments. For the qPCR experiments, the roots, stems, and leaves were collected during the vegetative growth stage of the plants. The samples from the different parts of the corolla for the qPCR were collected when the flowers were at F5 stage. All samples were collected and placed into Eppendorf tubes and then stored at −80°C for later analysis.

### RNA extraction, RT–PCR, and *PhENO1*, *PhPPT,* and *PhPPDK* gene cloning

Total RNA was isolated following the protocol described by Liu *et al.* [43]. The steps of reverse transcription are based on the steps of the commercially available kit from Vazyme. (R312, Vazyme, Nanjing, China). The full-length cDNA sequences of *PhENO1* (Peaxi162Scf00017g03139.1), *PhPPT1* (Peaxi162Scf00089g01842.1), *PhPPT2* (Peaxi162Scf00255g01119.1), and *PhPPDK* (Peaxi162Scf00095g00414.1) were obtained from *P. hybrida* ‘Ultra’ using specific primers (refer to Supplementary Table S1), designed based on sequences from the *Petunia axillaris* genome (Bombarely *et al.*, 2016). The full-length cDNA sequences of *PhENO1*, *PhPPT1*, *PhPPT2,* and *PhPPDK* were obtained from previously published genomic data (https://solgenomics.net/organism/Petunia_axillaris/genome) and specific primers were designed to amplify the full-length region from *P. hybrida* ‘Ultra’. The specific primers used for amplification were listed in Supplementary Table S1.

### Sequence analysis

Multiple sequence alignments were conducted, and a phylogenetic tree was constructed using DNAMAN and MEGA version 10.1.2 software. The identities of nucleotides and their corresponding translated amino acids were assessed through NCBI BLAST web service and TBtools [44]. Multiple sequence comparison and phylogenetic evolutionary tree construction were performed using DNAMAN and MEGA software. All nucleic acid sequences to be identified as well as their corresponding amino acid sequences were obtained using the National Center for Biotechnology Information (NCBI) BLAST and TBtools [44]. The InterPro software [45] along with NCBI CD-search was utilized to identify the conserved domains within the proteins. To achieve a deeper insight into the functions of PhENOs, PhPPTs, and PhPPDK, the protein sequences were analyzed using the MEME program to detect conserved motifs. The parameters set for this analysis included a range of zero to one for the number of repetitions and a cap of 10 motifs.

### Subcellular localization of PhENO1, PhPPT1, PhPPT2, and PhPPDK protein

The steps for subcellular localization experiments were performed as previously reported [37, 46, 47]. Full-length cDNA sequences of *PhENO1*, *PhPPT1*, *PhPPT2*, and *PhPPDK* were amplified via PCR and subsequently cloned into the pSAT-1403TZ vector, which contains GFP fusions under the control of the CaMV35S promoter. The primer sequences utilized for qPCR analysis are provided in Supplementary Table S2. Corolla and leaf protoplasts of *P. hybrida* ‘Ultra’ were used as transfection materials. The extraction of protoplasts was performed according to the following steps. First, corollas at the F5 stage and young leaves were selected and cut into fine strips using a blade. Subsequently, 2 ml of ISO buffer (0.34 M sorbitol, 20 mM KCl, 20 mM MES, and 10 mM CaCl_2_, adjusted to pH 5.7 with KOH) containing 0.07 g of an enzyme mixture (14.5 g/L Cellulase Onozuka R10, 2.6 g/L Macerozyme R-10, 8.7 g/L PVP-K30, and 8.7 g/L BSA) was added to the corollas and leaves. Enzymatic digestion was conducted for five hours and one hour at 28°C in corollas and leaves, respectively. After digestion, the corollas and leaves are collected, rinsed with ISO buffer, and centrifuged to obtain a suspension of protoplasts, which are then washed twice with 500 μl of W5 buffer (128 mM NaCl, 104 mM CaCl_2,_ 5 mM KCl, and 5 mM MES, adjusted to pH 5.7 with KOH). After washing with W5 buffer, the protoplasts were resuspended in an appropriate volume of MMG solution (0.3 M sorbitol, 16 mM MgCl_2_, 4 mM MES, adjusted to pH 5.7 with KOH). A total of 100 μl of protoplasts was used for each transformation, to which 10 μg of plasmid DNA was added, followed by 110 μl of polyethylene glycol solution [0.8 M sorbitol, 0.1 M Ca(NO_3_)_2_▪H_2_O, 40% polyethylene glycol adjusted to pH 8.0 with KOH]. Incubation at 25°C for 25 minutes was followed by washing with ISO buffer, as described previously, and the protoplasts are resuspended in 500 μl of W5 buffer after centrifugation. Due to the lack of chloroplasts in corolla protoplasts, a plastid-localized marker was added when using corolla protoplasts as the material for localization [35]. Fluorescence analysis occurred after a 24-hour dark incubation, with confocal microscopy performed using a Zeiss LSM800 microscope. The excitation/emission wavelengths for GFP and RFP were 488/535 nm and 488/637 nm, respectively.

### Quantitative real-time PCR assays

Quantitative real-time PCR (qPCR) assays were performed following established protocols [43]. *Cyclophilin* (*CYP*) (accession no. EST883944) served as the internal reference gene for quantifying cDNA abundance [37, 42]. The gene expression levels were determined and analyzed using the 2^-ΔΔCt^ method [48]. Primer sequences employed for qPCR analysis are detailed in Supplementary Table S2. Each treatment was assessed with three biological replicates, each comprising three technical replicates.

### Construction of pTRV vector and VIGS assay

The pTRV2-PhENO1, pTRV2-PhPPT, pTRV2-PhPPDK, pTRV2-PhENO1-PPT, pTRV2-PhENO1-PPDK, pTRV2-PhPPT-PPDK, and pTRV2-PhENO1-PPT-PPDK vectors were constructed by amplifying approximately 300 bp of *PhENO1* and *PhPPT* (the conserved regions of *PhPPT1* and *PhPPT2*) and *PhPPDK* using the specific primers listed in Supplementary Table S3 and cloning it into the pTRV2 vector. The resultant recombinant vector was transformed into the Agrobacterium tumefaciens strain GV3101 [49]. For the virus-induced gene silencing (VIGS) experiments, *P. hybrida* ‘Ultra’ at the transition from nutritive to reproductive growth stage were selected for infection, and 30 to 35 petunia seedlings were selected for infection in each group. After infection, the inoculated plants were first treated in dark conditions for 12 h, after which they were transferred to a greenhouse under controlled conditions as previously described.

### Total anthocyanin measurement

Petunia flowers were collected at Stage F5, and the corollas were harvested for analysis. Each sample consisted of 0.5 g of corolla and snap-frozen in liquid nitrogen. The extraction and quantification of anthocyanins were carried out as previously detailed [50]. Seven biological replicates, each consisting of three technical replicates, were analyzed for each treatment.

### Determination of free amino acids

We collected leaves from pTRV2-PhENO1-PPT-treated and control plants and ground them to powder with liquid nitrogen and pretreated them with 5% 5-sulfosalicylic acid dihydrate (Macklin, M327718069). The supernatant was taken for assay after centrifugation. The determination was carried out using Hitachi L-8900 amino acid analyzer according to the general rules of amino acid analysis method JY/T 0576-2020. The chromatographic column was Hitachi 855-4507 with column temperature of 135°C, detection wavelengths of 570 and 440 nm, elution pump flow rates of 0.35 ml·min^−1^, and derivatization pump flow rates of 0.30 ml·min^−1^. The external standards (FUJIFILLM, 010-28 164 and 016-28 144) method is used for quantification.

### Measurement of PEP, shikimate, and flavonoids content

The flowers at the blooming stage were collected from pTRV2-PhENO1-PPT-treated and control plants and then powdered them with liquid nitrogen. The supernatant was taken after centrifugation for assay. The PEP, shikimate and flavonoids content were determined by the enzyme-linked immunosorbent assay (ELISA) Kits (MEIMIAN, Suzhou, China) following the manufacturer’s instructions. The kits and catalog numbers are as follows: PEP (MM-47160O1), shikimate (MM-62849O2), and flavonoids (MM-205301). The absorbance was collected by using Thermo Scientific Multiskan SkyHigh (A51119600DPC). Each treatment was assessed with three biological replicates, each comprising five technical repeats.

### Statistical analysis

The statistical analysis used one-way ANOVA followed by Duncan’s multiple range test with at least three replicates. *P*-values ≤0.05 were considered significant.

## Supplementary Material

Web_Material_uhaf040

## Data Availability

All data included in this study are included in this article and its supplementary information files.
