# Supplementary material for: Co-silencing of PhENO1 and PhPPT alters anthocyanin production by reducing phosphoenolpyruvate supply in petunia flower
Source: Hortic Res. 2025 Feb 11;12(5):uhaf040. doi: 10.1093/hr/uhaf040 (PMC11997433; doi:10.1093/hr/uhaf040)
Supplement: Web_Material_uhaf040 [file web_material_uhaf040.zip › Supplementary file.docx]

**Supplementary file**


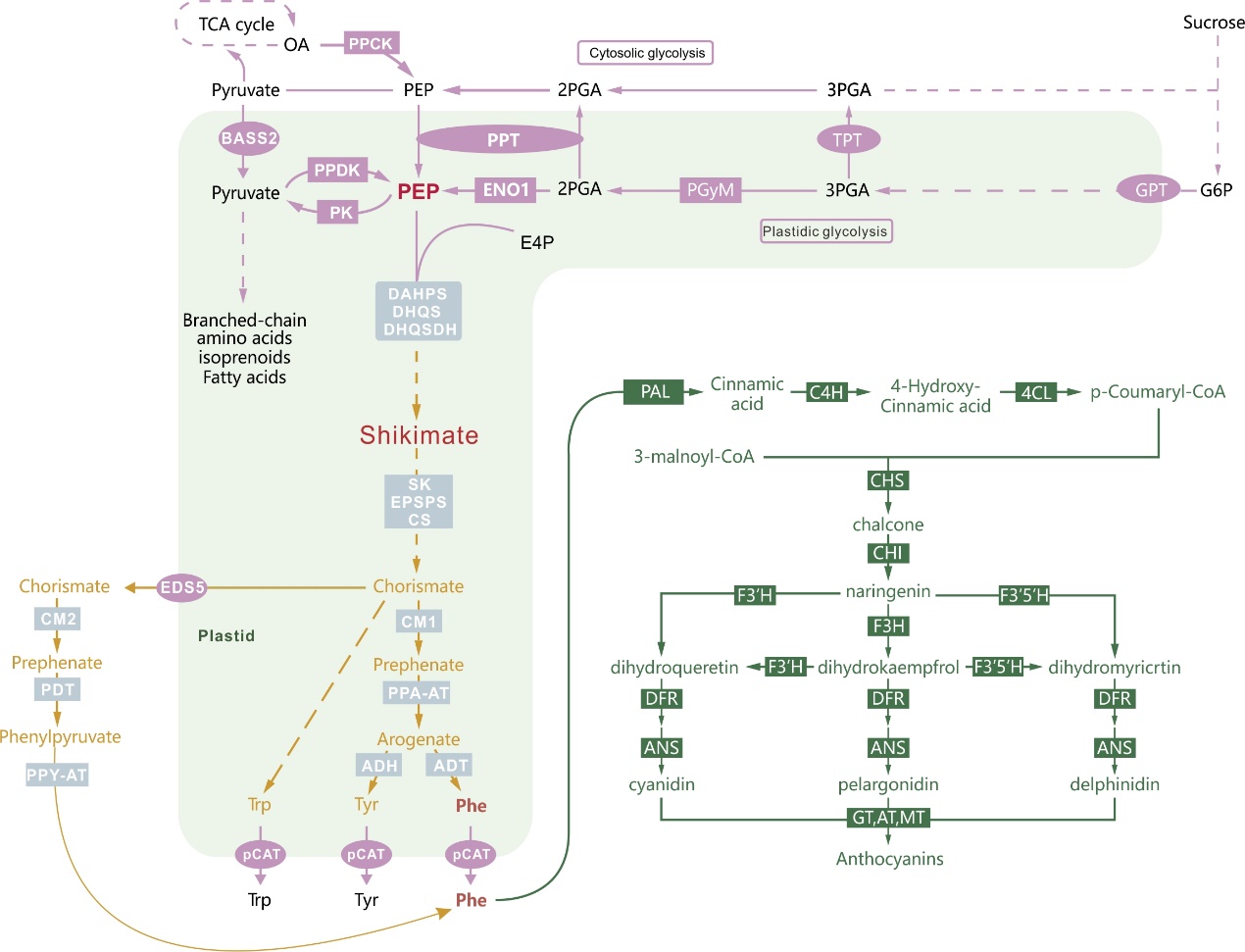


**Fig. S1** Diagram of three ways to form plastidic phosphoenolpyruvate (PEP) modified from Maeda & Dudareva (2012). (1) PEP can be produced in the plastid through the complete glycolytic pathway catalyzed by phosphoglyceromutase (PGyM) and enolase (ENO). The conversion of 3-phosphoglycerate (3PGA) to 2-phosphoglycerate (2PGA) is facilitated by PGYM, followed by the conversion of 2PGA to PEP catalyzed by ENO1. (2) 3PGA can be transported to the cytoplasm through the triose phosphate (TPT) and generate PEP in the cytoplasm, PEP is further transported into plastid through phosphoenolpyruvate/phosphate translocator (PPT). (3) Pyruvate can be catalyzed by pyruvate orthophosphate dikinase (PPDK) to form PEP in plasid, PEP can also produce pyruvate through the catalysis of pyruvate kinase (PK). End products of the shikimate pathway are three aromatic amino acids (AAA), phenylalanine (Phe), tryptophan (Trp) and tyrosine (Tyr). Phe can be used as the precursors to synthesis anthocyanins. E4P, erythrose-4-phosphate; DAHPS, 3-Deoxy-D-arabino-heptulosonate 7-phosphate synthase; DHQS, 3-Dehydroquinate synthase; DHQSDH, 3-Dehydroquinate dehydratase and shikimate dehydrogenase; SK, shikimate kinase; EPSPS, 5-Enolpyruvylshikimate 3-phosphate synthase; CS, chorismate synthase; CM, chorismate Mutase; PDT, prephenate dehydratase; PPY-AT, phenylpyruvate aminotransferase; pCAT, plastidial cationic amino-acid transporter; BASS2, bile acid:sodium symporter family protein 2; PAL, phenylalanine ammonia lyase; C4H, cinnamate4-hydroxy lase; 4CL, 4-coumarate CoA ligase; CHS, chalcone synthase; CHI, chalcone isomerase; F3’H, flavanone 3’-hydroxylase; F3’5’H, flavanone 3’5’-hydroxylase; F3H, flavanone3-hydroxylase; DFR, dihydroflavonol 4-reductase; ANS, anthocyanidin synthase; GT, glucosyltransferase; AT, acyltransferase; MT, methyl-transferase.


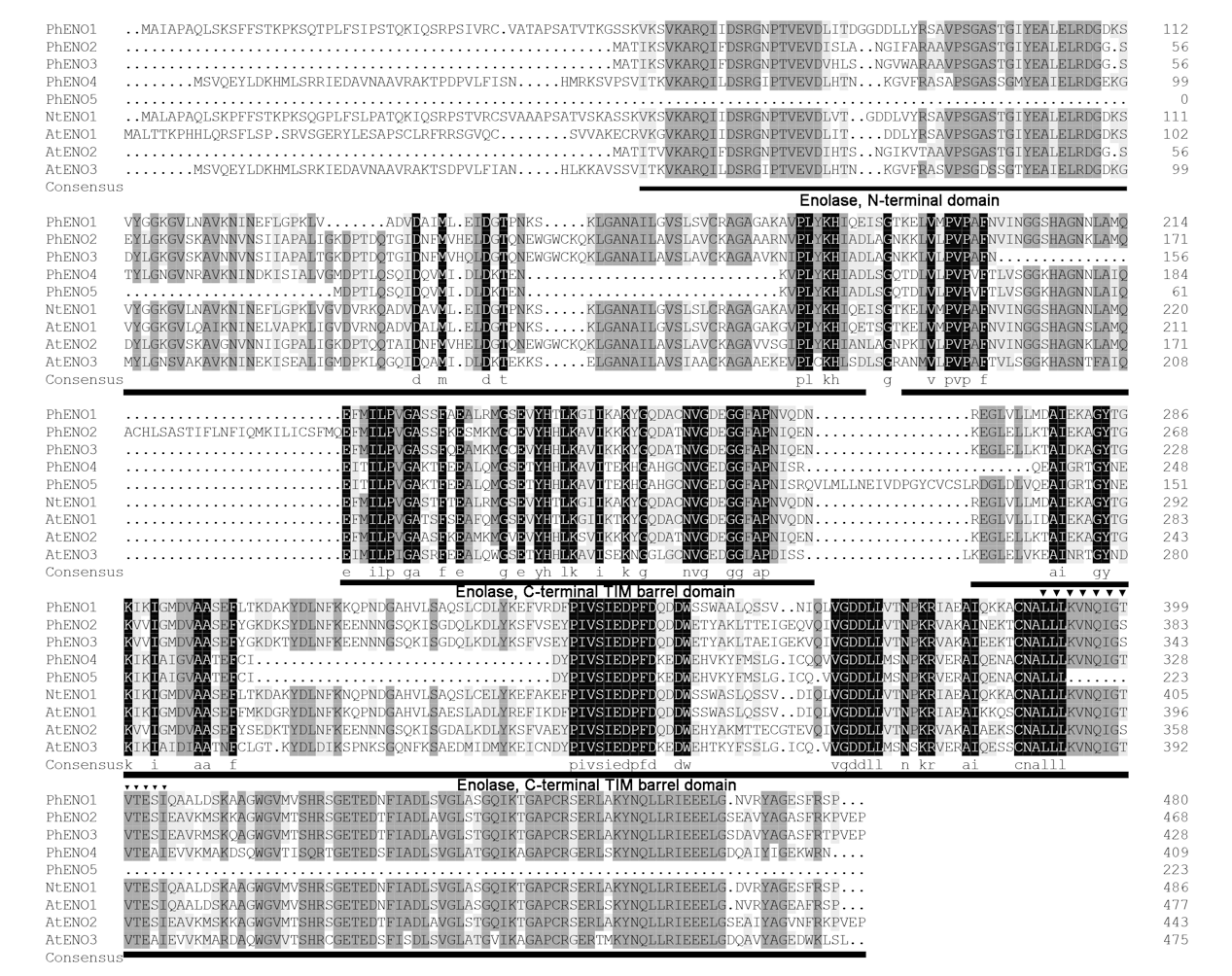


**Fig. S2** Multiple alignment of deduced amino acid sequences of ENOs. Alignment among the deduced amino acid sequences of ENO genes in plants was carried out using DNAMAN software. Gene ID were as follow: PhENO1, Peaxi162Scf00017g03139.1; PhENO2, Peaxi162Scf00433g00096.1; PhENO3, Peaxi162Scf00110g01814; PhENO4, Peaxi162Scf01058g00157.1; PhENO5, Peaxi162Scf71174g00002.1 NtENO1, XP_016458052.1; AtENO1, AT1G74030.1; PfENO1, KAH6807707.1. Black shading indicates amino acid residues conserved among all ENOs. Gray shading indicates only some of amino acids are the same. The underline indicates two conserved domains among ENOs, enolase, N-terminal domain (InterPro ID, IPR020811) and enolase, C-terminal TIM barrel domain. Inverted triangle indicates conserved site among ENOs.


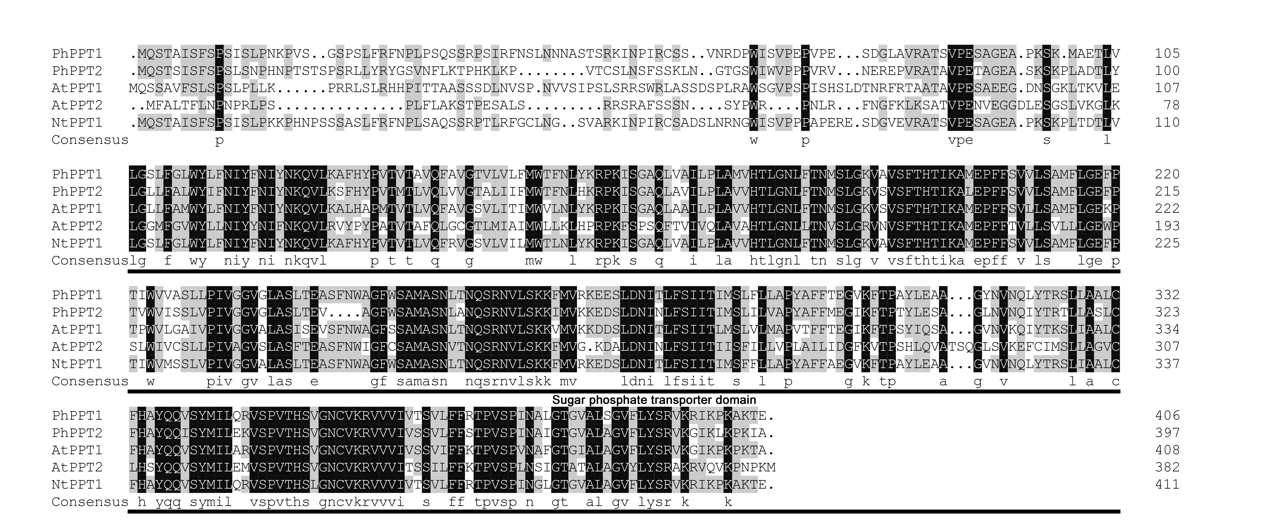


**Fig. S3** Multiple alignment of deduced amino acid sequences of PPTs. Alignment among the deduced amino acid sequences of PPT genes in plants was carried out using DNAMAN software. Gene ID were as follow: PhPPT1, Peaxi162Scf00089g01842.1; PhPPT2, Peaxi162Scf00255g01119.1; AtPPT1, AT5G33320.1; AtPPT2, At3g01550; NtPPT1, NP_001312810.1. Black shading indicates amino acid residues conserved among all PPTs. Gray shading indicates only some of amino acids are the same. The underline indicates conserved domain among PPTs, sugar phosphate transporter domain (InterPro ID, IPR004853).


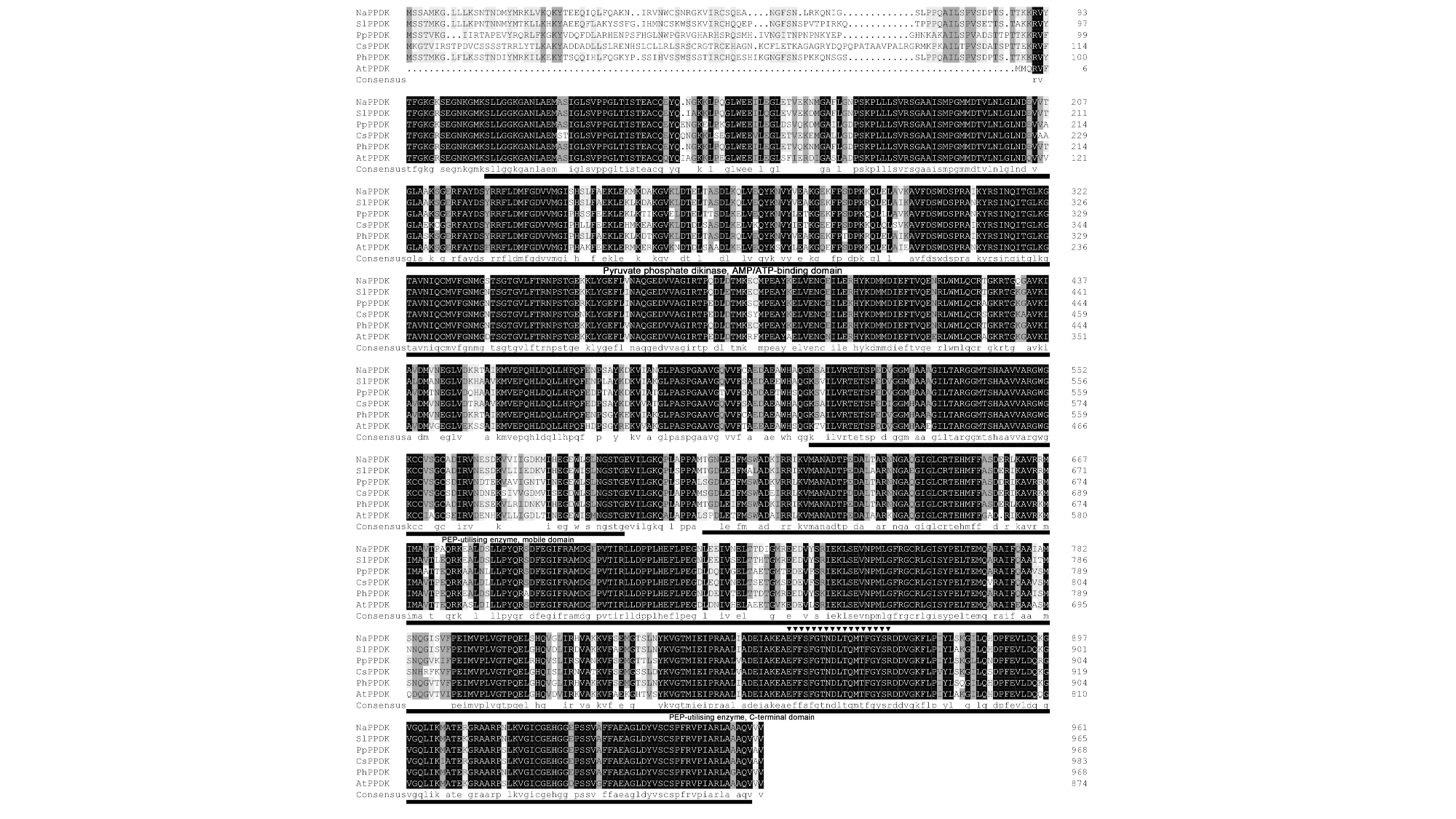


**Fig. S4** Multiple alignment of deduced amino acid sequences of PPDKs. Alignment among the deduced amino acid sequences of PPT genes in plants was carried out using DNAMAN software. Gene ID were as follow: NaPPDK, XP_019259950.1; SlPPDK, XP_010322950.1; PpPPDK, XP_007225359.1; CsPPDK, KAH9683120.1; PhPPDK, Peaxi162Scf00095g00414.1; AtPPDK, At4g15530. Black shading indicates amino acid residues conserved among all PPDKs. Gray shading indicates only some of amino acids are the same. The underline indicates three conserved domains among PPDKs, Pyruvate phosphate dikinase, AMP/ATP-binding domain (InterPro ID, IPR002192), PEP-utilising enzyme, mobile domain (InterPro ID, IPR008279) and PEP-utilising enzyme, C-terminal domain (InterPro ID, IPR000121). Inverted triangle indicates conserved site among PPDKs.


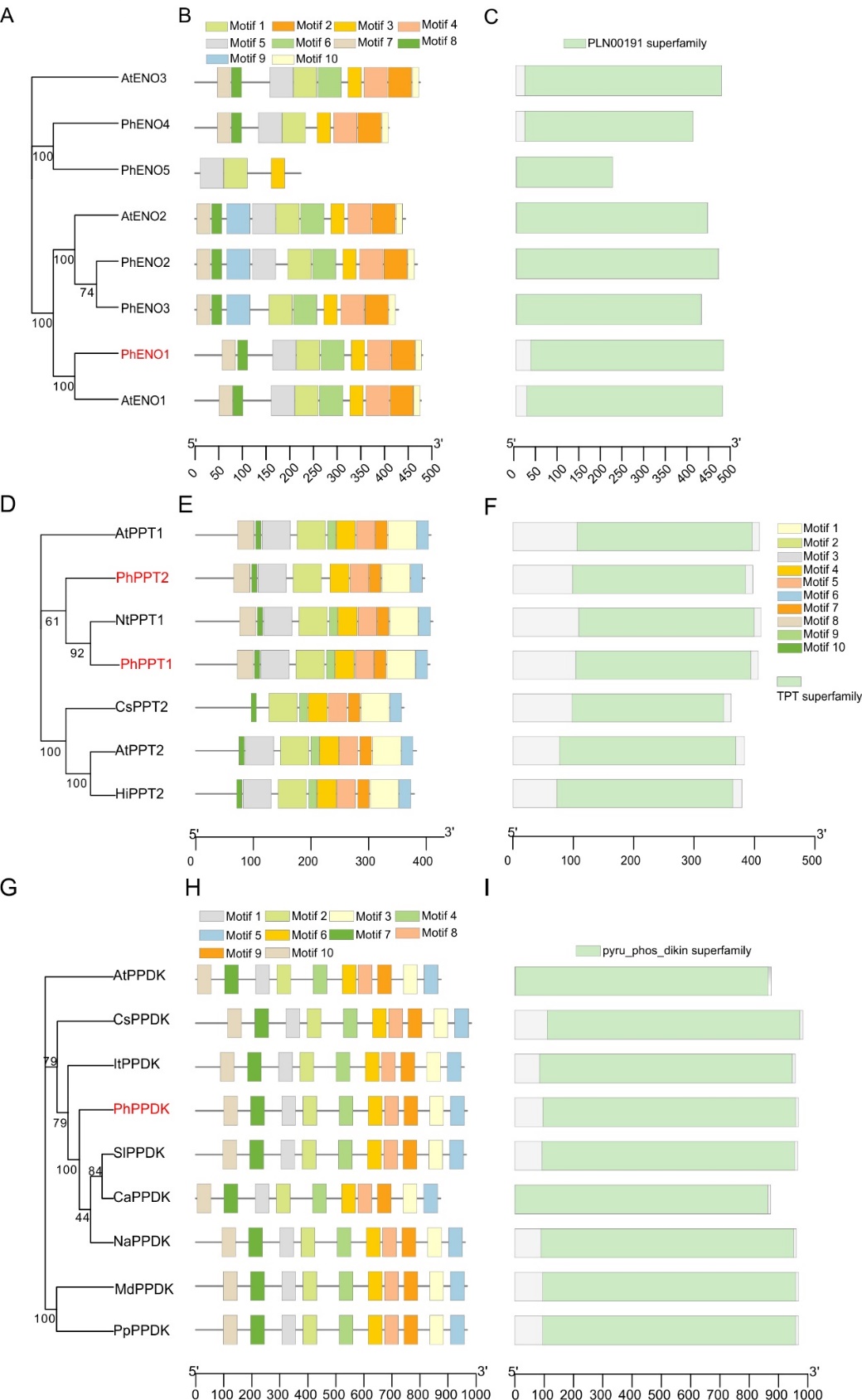


Fig. S5 Phylogenetic tree, motifs and protein conserved structural domains of analyses of ENO, PPT and PPDK members from different species.

A Neighbor-Joining (NJ) phylogenetic trees of ENO (A), PPT (D) and PPDK (G) families were constructed by MEGA7.0 with 1000 bootstrap replicates. The GenBank/EMBL accession numbers for the sequences used in the phylogenetic trees are showed in Supplementary table S5. The different color boxes in (B), (E) and (H) means different motifs in ENO, PPT and PPDK families. Green box means the conserved domain in ENO (C), PPT (F) and PPDK (I) families.


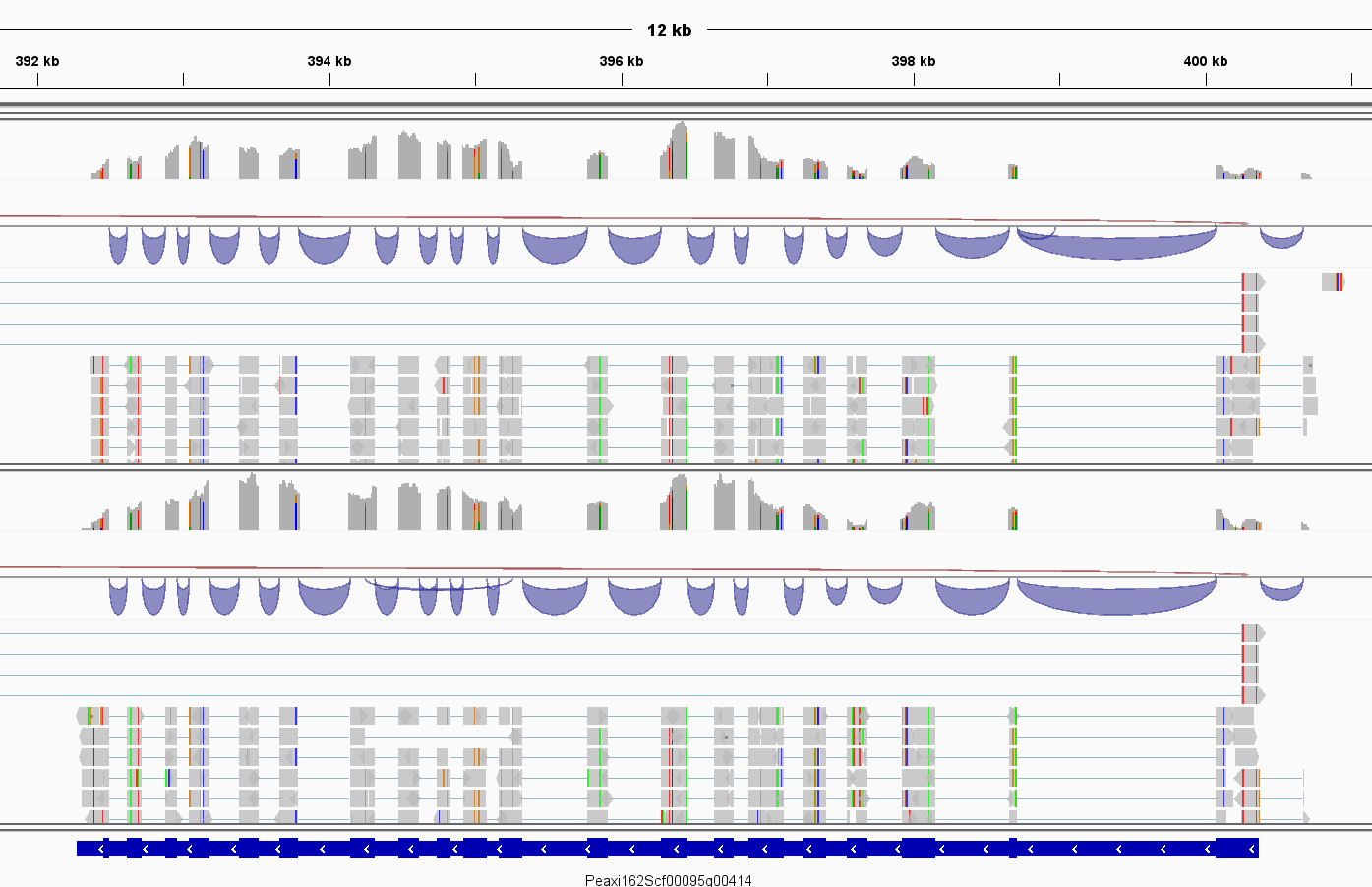


Fig. S6 Gene structure analysis of *PhPPDK*

The gene structure of the *PhPPDK* gene under the petunia corolla transcriptome (Zhao et al., 2020) was demonstrated by IGV software. The transcriptome data support the existence of only one transcript for this gene without alternative splicing events.


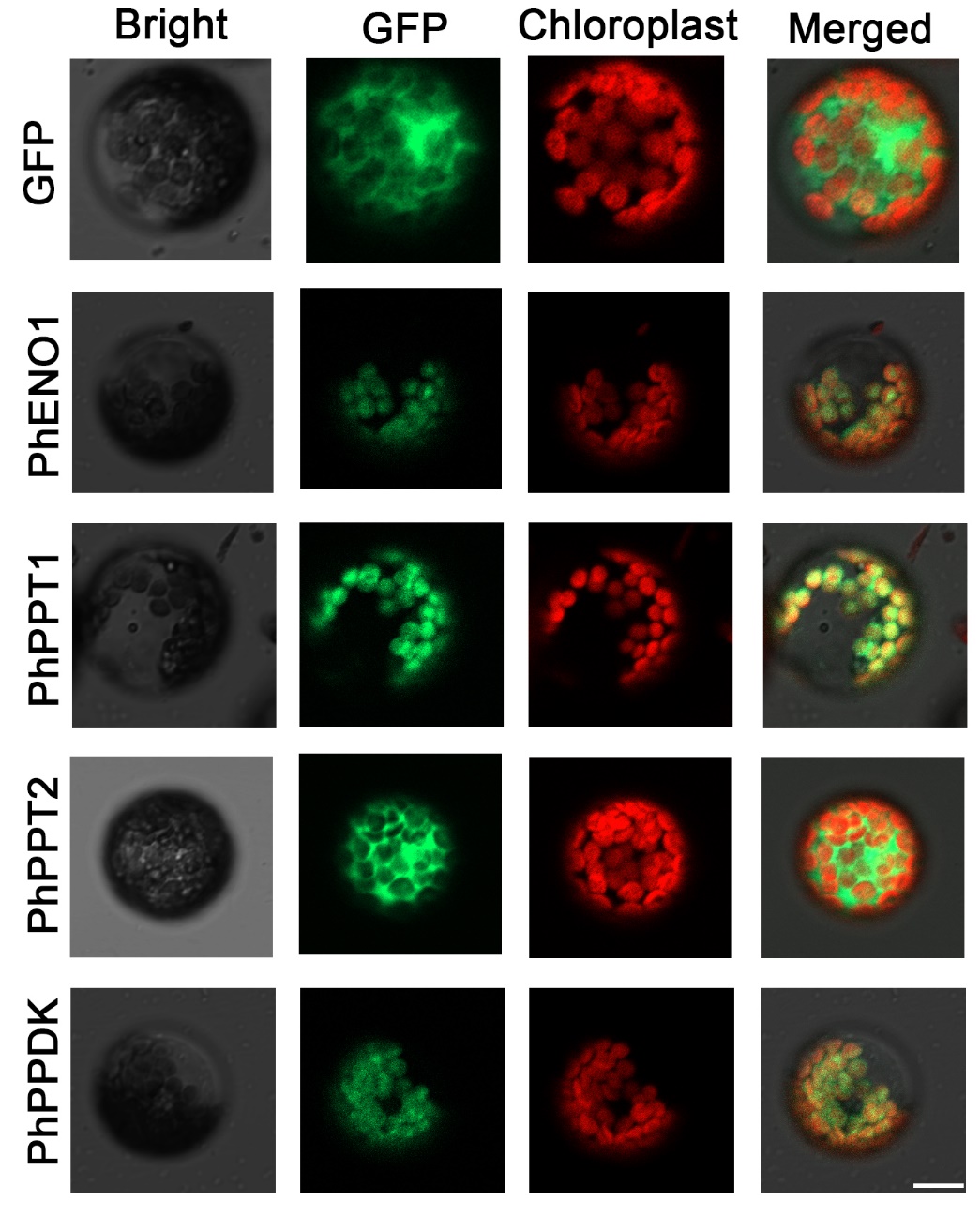


Fig. S7 Subcellular localization of free-GFP, PhENO1, PhPPT1, PhPPT2 and PhPPDK in leaf protoplasts. Bars = 10 μm.


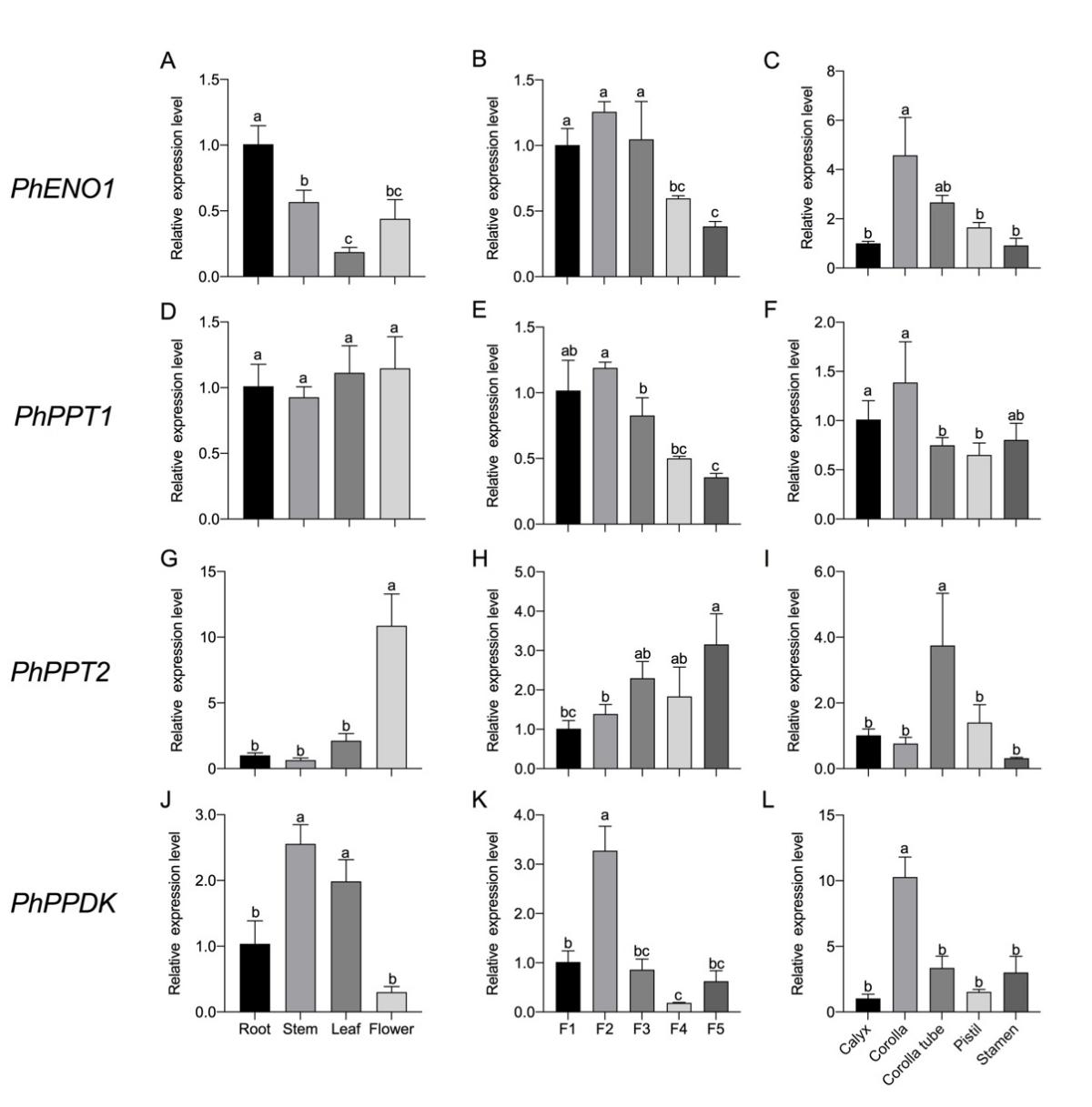


**Fig. S8** The expression patterns of *PhENO1*, *PhPPT1, PhPPT2* and *PhPPDK* determined using quantitative real-time PCR. Expression of *PhENO1* (A), *PhPPT1* (D), PhPPT2 (G) and *PhPPDK* (J) in different organs (root, stem, leaf and flower). Expression of *PhENO1* (B), *PhPPT1* (E), PhPPT2 (H) and *PhPPDK* (K) in different flower development. Flower development was divided into five stages: F1 (length 0.5 cm), F2 (1.0 cm), F3 (2.0 cm, the coloring stage of flower), F4 (3.0 cm), F5 (flowering stage). Expression of *PhENO1* (C), *PhPPT1* (F), PhPPT2 (I) and *PhPPDK* (L) in different flower parts (corolla,corolla tube, stamen, pistil and calyx). *CYCLOPHILIN* (accession no. EST883944) was used as the internal reference gene to quantify the cDNA abundance. Data are presented as the mean ± SD (n = 3). Statistical analysis was performed using one-way analysis of variance (ANOVA) followed by Duncan's multiple range test (DMRT) with three biological replicates.

**
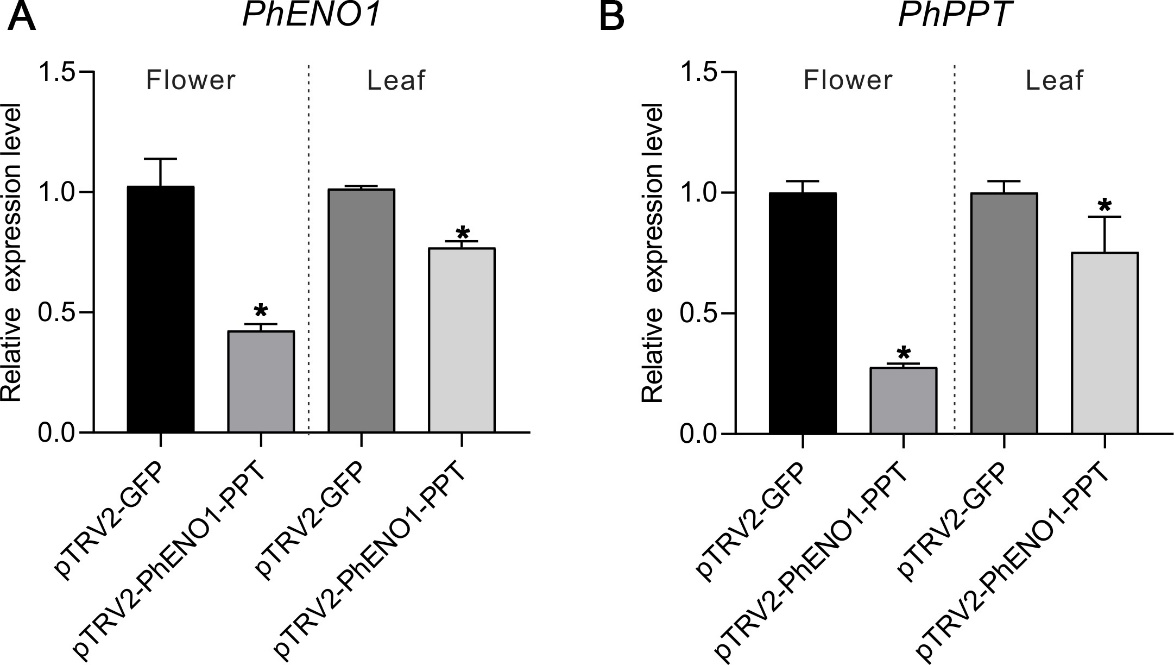
**

Fig. S9 The expression levels of *PhENO1* (A) and *PhPPT* (B) in the flowers and leaves of pTRV2-PhENO1-PPT treated plants. Data are presented as mean ± SD (n = 3). An asterisk means significant difference at p < 0.05 level.


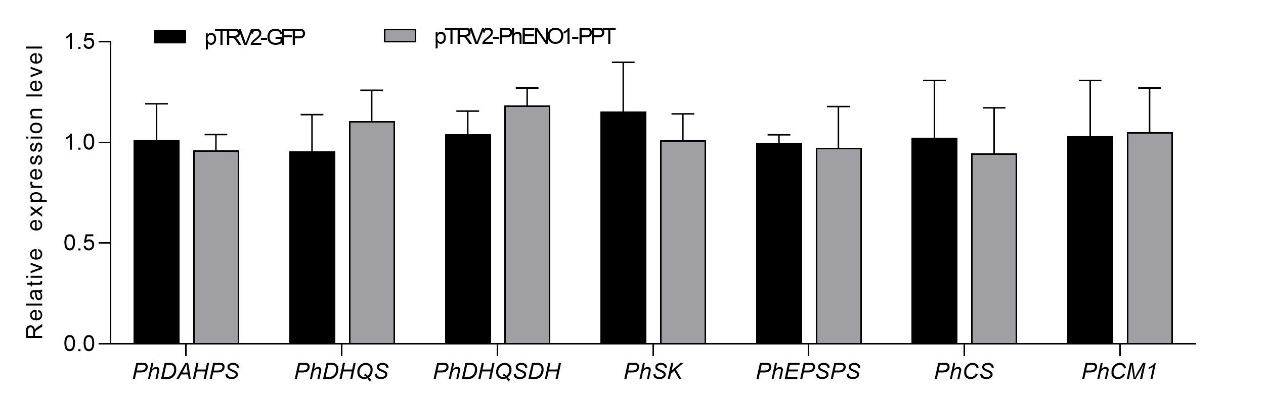


Fig. S10 Expression levels of 6 key genes in shikimate pathway in PTRV2-PhENO1-PPT treated plants and control determined by quantitative real-time PCR. Data are presented as the mean ± SD (n = 3). Statistical analysis was performed using Student’s t test with three biological replicates.


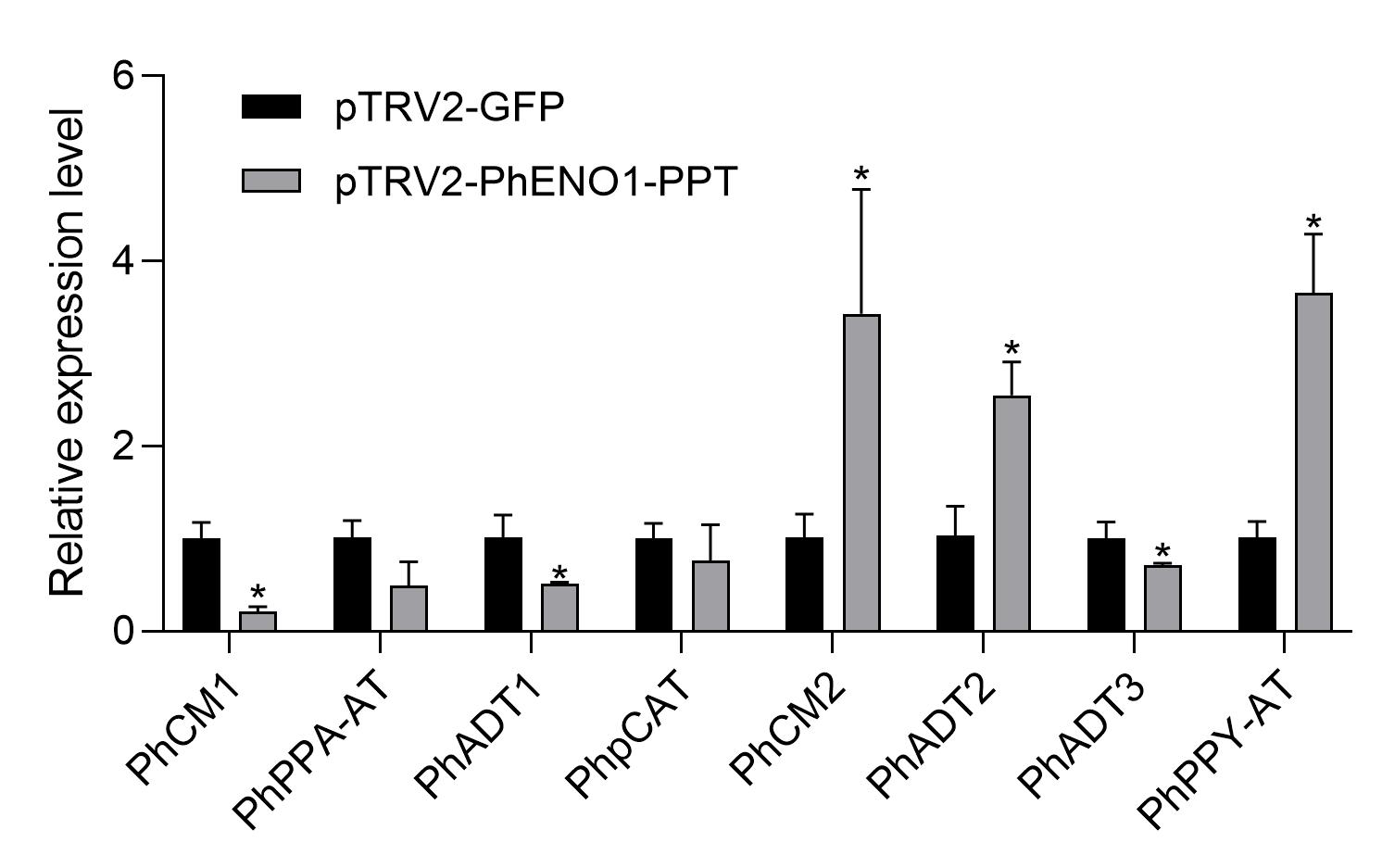


Fig. S11 Expression levels of 8 key genes in phenylalanine pathway in PTRV2-PhENO1-PPT treated plants and control determined by quantitative real-time PCR. Data are presented as the mean ± SD (n = 3). Statistical analysis was performed using Student’s t test with three biological replicates.


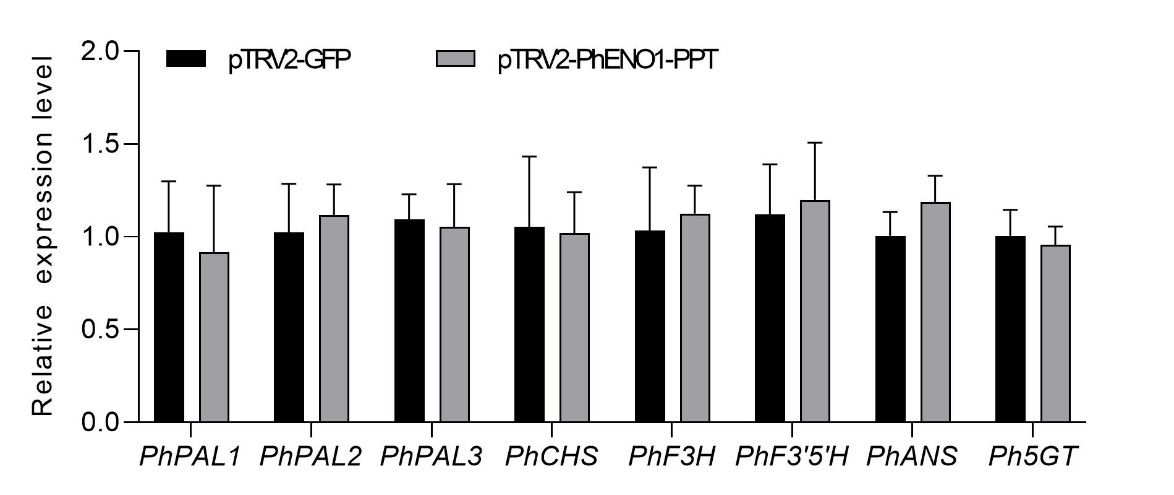


Fig. S12 Expression levels of 8 key genes in anthocyanin pathway in PTRV2-PhENO1-PPT treated plants and control determined by quantitative real-time PCR. Data are presented as the mean ± SD (n = 3). Statistical analysis was performed using Student’s t test with three biological replicates.

Table.S1 Primer sequences used in RT-PCR

| Primer name | Sequence |
| --- | --- |
| PhENO1-F | ATGGCCATAGCTCCAGCTCAA |
| PhENO1-R | TCAAGGAGATCTGAAAGATTCACCA |
| PhENO2-F | ATGGCAACTATCAAATCTGTTAAGGC |
| PhENO2-R | TTAGTAGGGCTCAACAGGCTT |
| PhENO3-F | ATGGCTACTATTAAATCCGTTAAGGC |
| PhENO3-R | TTAGTAGGGCTCAACAGGTGTAC |
| PhENO4-F | ATGTCAGTACAAGAATATCTAGATAA |
| PhENO4-R | TCAATTCCTCCACTTTTCACCAATAT |
| PhENO5-F | ATGGATCCAACTCTTCAATCTCAG |
| PhENO5-R | AAGAAGAAGGGCGTTGCAC |
| PhPPT1-F | ATGCAAAGCACAGCTATTTCA |
| PhPPT1-R | TCATTCTGTTTTTGCCTTAGGC |
| PhPPT2-F | ATGCAAAGCACATCAATCTCATTC |
| PhPPT2-R | TCAAGCTATCTTTGGCTTCAGTTTG |
| PhPPDK-F | ATGGCCATAGCTCCAGCTCAA |
| PhPPDK-R | TCAAGGAGATCTGAAAGATTCACCA |

Table.S2 Primer sequences used in subcellular localization

| Primer name | Sequence |
| --- | --- |
| PhENO1-F | TCACCATTTACGAACGATAGCCATGGCAATGGCCATAGCTCCAGCT |
| PhENO1-R | AGCTCCTCGCCCTTGCTCACCATGGAAGGAGATCTGAAAGATTCACCAGC |
| PhPPT1-F | TCACCATTTACGAACGATAGCCATGGCA ATGCAAAGCACAGCTATTTCATTTTC |
| PhPPT1-R | AGCTCCTCGCCCTTGCTCACCATGGATTCTGTTTTTGCCTTAGGCTTAA |
| PhPPT2-F | ACCATTTACGAACGATAGCCATGGTGATGCAAAGCACATCAATCTCATTCT |
| PhPPT2-R | CTCCTCGCCCTTGCTCACCATGGCAGCTATCTTTGGCTTCAGTTTGAT |
| PPDK-F | ACCATTTACGAACGATAGCCATGGTGATGAGTTCAACTATGAAGGGGTTG |
| PPDK-R | CTCCTCGCCCTTGCTCACCATGGCAACTACAACTTGAGCTCCAGC |

Table.S3 Primer sequences used in RT-qPCR

| Primer name | Sequence |
| --- | --- |
| qRT-PhENO1-F | GTGATGAAGGTGGATTCGCTC |
| qRT-PhENO1-R | GGAACTCAGACGCTGCTACATC |
| qRT-PhPPT1-F | AGTTTGTTGTCCATGTTAGACGC |
| qRT-PhPPT1-R | TCAACGCACATATGCTGCAAT |
| qRT-PhPPT2-F | CGCTCGGTGGTAGATTAG |
| qRT-PhPPT2-R | AGTAACGAGACGTGAATGATAAC |
| qRT-PhPPDK-F | GGACGAGATTGTCACTGAACTAAC |
| qRT-PhPPDK-R | CTGAAAGATTGCACGAGCCT |
| qRT-PhCS-F | TTCCTTTCTCGCCCTTCA |
| qRT-PhCS-R | CAACCAACTCCACCACCA |
| qRT-PhSK-F | TATTTACTTTCTTGACGAGGAG |
| qRT-PhSK-R | TTGCTGGATGTTCATTCTTA |
| qRT-PhDAHPS-F | AAGGAAGCCACCCAGGAG |
| qRT-PhDAHPS-R | GGGATTGAGATGCGTTGA |
| qRT-PhDHQSDH-F | ACCAAGGGTTGATGATAC |
| qRT-PhDHQSDH-R | CCCGCAAGAGTCTAGTGA |
| qRT-PhDHQS-F | TTATGGCACAGGTAGATTC |
| qRT-PhDHQS-R | CTGGTAAAGTATTCAGGGT |
| qRT-PhEPSPS-F | GGAGCAACCGTTGAAGAAGG |
| qRT-PhEPSPS-R | AGCAGCAAGAGAAAAAGCCA |
| qRT-PhCM1-F | TCAACATTGAGGTGAGAAAGAGCT |
| qRT-PhCM1-R | CACTCTTCTACACTCTCAGGGACTC |
| qRT-PhPPA-AT-F | TCCGTATCTCCTATGCAGCATC |
| qRT-PhPPA-AT-R | GAGGGCGAGACTACGAACTATAAGG |
| qRT-PhADT1-F | TAACTGCGAAGCCATTCCCTGC |
| qRT-PhADT1-R | CTCTACTGGTAGAACTGCGCG |
| qRT-PhpCAT-F | AGCACTTTCCGATACCCCAA |
| qRT-PhpCAT-R | GAACTCGGCTTGTTTCTTCGA |
| qRT-PhCM2-F | TGCAACTACTGCTGCCTGTGAT |
| qRT-PhCM2-R | TCGTCAGAGCAATCCCTGAAT |
| qRT-PhADT2-F | ACGAAGTTGGGTTTGGTCAG |
| qRT-PhADT2-R | TGCCCCTGCATCTTTTAGTT |
| qRT-PhADT3-F | CAAAATGTGAAGCTATTCCTTGTG |
| qRT-PhADT3-R | TTCGATCGGTAAAACAGCACG |
| qRT-PhPPY-AT-F | GCCTTGTCAATTCTGGCTCG |
| qRT-PhPPY-AT-R | CAGCATTGAGATCAACCTCCC |
| qRT-PhPAL1-F | ATAGCTAGCCAATCGGGCAC |
| qRT-PhPAL1-R | CCGTTGCCAAAGATTCCAGC |
| qRT-PhPAL2-F | TGCCTGCAGTGCTAACTACC |
| qRT-PhPAL2-R | CACGGCCACTAACTCGTCTT |
| qRT-PhPAL3-F | TGGGCTTAATCTCGGCAAGG |
| qRT-PhPAL3-R | CGGAGCAACTCCTTCTCACA |
| qRT-PhCHS-F | ACATGGCACCTTCTCTTGATG |
| qRT-PhCHS-R | GGTAATTTTGGACTTGGGCTG |
| qRT-PhF3H-F | GCCTTAACCAAGGCATGTGT |
| qRT-PhF3H-R | TAGCTTGAAGCCCACCAACT |
| qRT-PhF3’5’H-F | CAACTTCTCCAATCGTCCACC |
| qRT-PhF3’5’H-R | CCTTAGCAACTTCCATCGTGG |
| qRT-PhANS-F | TCTTCCATTGTGCTTTCCCTG |
| qRT-PhANS-R | GTTGCTGGAGTGTAGTCAGTAG |
| qRT-Ph5GT-F | GCCAGTTGTGGCATTTCCTC |
| qRT-Ph5GT-R | CCTTTCTCTCCTCCATCCATTAC |
| qRT-PhCYP-F | AGGCTCATCATTCCACCGTGT |
| qRT-PhCYP-R | TCATCTGCGAACTTAGCACCG |

Table.S4 Primer sequences used in VIGS

| Primer name | Sequence |
| --- | --- |
| pTRV2-ENO1-F | GTGAGCTCGGTACCGGATCCGGGCAGCACTACAATCTTCAG |
| pTRV2-ENO1-R | TGAGTAAGGTTACCGAATTCCACCAGCATAGCGAACATTTCC |
| pTRV2-PPT1-F | GTGAGCTCGGTACCGGATCCATGGGTTGTGGCTTCTCTTC |
| pTRV2-PPT1-R | TGAGTAAGGTTACCGAATTCGCTGCAAGGAGAGACCTAGTG |
| pTRV2-PPDK-F | GTGAGCTCGGTACCGGATCCCAGAAATGCAGGCTCGTG |
| pTRV2-PPDK-R | TGAGTAAGGTTACCGAATTCCTTGCCTACATCGTCTCTGC |
| pTRV2-ENO1-PPT-1-F | GTGAGCTCGGTACCGGATCCGGGCAGCACTACAATCTTCAGTT |
| pTRV2-ENO1-PPT-1-R | CACAACCCATCACCAGCATAGCGAACATTTCC |
| pTRV2-ENO1-PPT-2-F | TATGCTGGTGATGGGTTGTGGCTTCTCTTCTACC |
| pTRV2-ENO1p-PPT-2-R | TGAGTAAGGTTACCGAATTCGCTGCAAGGAGAGACCTAGTGTAA |
| pTRV2-ENO1-PPDK-1-F | GTGAGCTCGGTACCGGATCCGGGCAGCACTACAATCTTCAGTT |
| pTRV2-ENO1-PPDK-1-R | CTGCATTTCTGCACCAGCATAGCGAACATTTCC |
| pTRV2-ENO1-PPDK-2-F | ATGCTGGTGCAGAAATGCAGGCTCGTGCA |
| pTRV2-ENO1-PPDK-2-R | TGAGTAAGGTTACCGAATTCCTTGCCTACATCGTCTCTGCTATATC |
| pTRV2-PPT-PPDK-1-F | GTGAGCTCGGTACCGGATCCATGGGTTGTGGCTTCTCTTCTACC |
| pTRV2-PPT-PPDK-1-R | TGCATTTCTGGCTGCAAGGAGAGACCTAGTGTAA |
| pTRV2-PPT-PPDK-2-F | TCCTTGCAGCCAGAAATGCAGGCTCGTGCA |
| pTRV2-PPT-PPDK-2-R | TGAGTAAGGTTACCGAATTCCTTGCCTACATCGTCTCTGCTATATC |
| pTRV2-ENO1-PPT-PPDK-1-F | GTGAGCTCGGTACCGGATCCGGGCAGCACTACAATCTTCAGTT |
| pTRV2-ENO1-PPT-PPDK-1-R | CACAACCCATCACCAGCATAGCGAACATTTCC |
| pTRV2-ENO1-PPT-PPDK-2-F | TATGCTGGTGATGGGTTGTGGCTTCTCTTCTACC |
| pTRV2-ENO1-PPT-PPDK-2-R | TGCATTTCTGGCTGCAAGGAGAGACCTAGTGTAA |
| pTRV2-ENO1-PPT-PPDK-3-F | TCCTTGCAGCCAGAAATGCAGGCTCGTGCA |
| pTRV2-ENO1-PPT-PPDK-3-R | TGAGTAAGGTTACCGAATTCCTTGCCTACATCGTCTCTGCTATATC |
